# Significance of a Three-Missense Pathogenic Variant in the Substrate-Binding Lesion in a Subject With 21-Hydroxylase Deficiency: A Case Report

**DOI:** 10.7759/cureus.97470

**Published:** 2025-11-21

**Authors:** Ayaka Harada, Takatoshi Anno, Hayato Isobe, Kohei Kaku, Hideaki Kaneto

**Affiliations:** 1 Diabetes, Metabolism, and Endocrinology, Kawasaki Medical School, Kurashiki, JPN; 2 Diabetes and Endocrinology, Kurashiki Central Hospital, Kurashiki, JPN; 3 General Internal Medicine 1, Kawasaki Medical School, Kurashiki, JPN

**Keywords:** 21-hydroxylase, 21-hydroxylase deficiency, cluster mutation, congenital adrenal hyperplasia, salt-wasting form

## Abstract

Congenital 21-hydroxylase deficiency (21-OHD) represents a healthcare challenge during the transition from childhood to adulthood for young adults with special needs. Although numerous countries have implemented newborn screening programs for 21-OHD, a significant number of patients may reach adulthood without undergoing genetic testing. In this report, we present a case of a 26-year-old Japanese woman with 21-OHD, who was referred to our hospital for the transition from childhood to adulthood in young adults with special healthcare needs (YASHCN). At that time, she had no complaints, but she wanted to undergo a thorough examination for a correct diagnosis of 21-OHD. The genetic test showed a novel new variant of the three-cluster mutation, p.[Ile237Lys; Val238Glu; Met240Lys]. Our findings highlight the significance and reaffirmation of the three-missense pathogenic variant in the CYP21A2 gene in determining the enzyme activity of 21-hydroxylase and the onset of 21-OHD.

## Introduction

The 21-hydroxylase deficiency (21-OHD) is a congenital condition that requires a seamless transition from childhood to adulthood for young adults with special healthcare needs (YASHCN). Healthcare transition in YASHCN aims to promote lifelong functioning and potential through the delivery of appropriate healthcare services that are uninterrupted as the individual advances [1]. Meanwhile, various countries have established newborn screening programs for 21-OHD at birth that involve the assessment of symptoms and measurement of 17-hydroxyprogesterone concentration. If a positive result is obtained from the newborn screen test for this condition, further biochemical and genetic tests are carried out to confirm the diagnosis [2]. As a result, some 21-OHD patients may reach adulthood without having undergone genetic testing.

Congenital adrenal hyperplasia (CAH) is a group of rare autosomal recessive disorders characterized by a reduction in enzyme activity involved in adrenal steroidogenesis, resulting in impaired cortisol synthesis in the adrenal gland [3-6]. CAH is a widespread adrenal event, and most of the causative diseases of CAH are 21-OHD, but there are others, such as P-450 oxidoreductase deficiency, 11β-hydroxylase deficiency, and others. The 21-hydroxylase gene (CYP21A2), which encodes the adrenal steroidogenic enzyme, is located on the long arm of chromosome 6 within the major histocompatibility complex (MHC) [7]. Mutations in CYP21A2 are responsible for approximately 95%-99% of all CAH cases [5,8,9]. 21-OHD has been classified into three main forms: (1) the salt-wasting or salt-losing form (SW), (2) the simple virilizing form (SV), and (3) the non-classic or late-onset form (NC). The SW and SV forms of 21-OHD are considered the "classic" forms, while NC is the most common form of 21-OHD [10]. Approximately 67% of all classic 21-OHD cases are classified as SW, while the remainder are SV [11]. 21-OHD is a rare disease, with most studies indicating a frequency of 1:14,000 to 1:18,000 live births [12].

In this report, we present a case of a newly discovered three-missense pathogenic variant in the subject with 21-OHD. This cluster of three missense pathogenic variants in the G helix also completely abolishes enzymatic activity, possibly by interfering with substrate binding, as previously reported for a similar three-missense pathogenic variant [13]. We believe that it is important for clinicians to understand this subject because this case emphasizes the importance of understanding the correct gene mutation, as the treatment strategy depends on the degree of residual activity of 21-hydroxylase in the 21-OHD patients, and misclassification can lead to suboptimal treatment.

## Case presentation

A 26-year-old Japanese woman was referred to our hospital for transition from childhood to adulthood in YASHCN. She was diagnosed with 21-OHD due to poor feeding, vomiting, and ambiguous genitalia at birth, as well as elevated 17α-hydroxyprogesterone levels. She had undergone vulvoplasty several times during her childhood. On the other hand, she was diagnosed as an SW type and treated with glucocorticoid and mineralocorticoid replacement therapy at that time. She was taking 25 mg/day of hydrocortisone and 0.1 mg/day of fludrocortisone for the treatment, and 100 mg of hydrocortisone was infused when needed. She had no complaints because she was referred to our hospital for the transition from childhood to adulthood in YASHCN, and her symptoms were short stature and hyperpigmentation. Her height and body weight were 145.3 cm and 47.1 kg, respectively. Her vital signs at the reference to our hospital were as follows: temperature, 36.7°C; blood pressure, 117/63 mmHg; heart rate, 60 beats/min; oxygen saturation, 98% (room air). As shown in Table 1, laboratory data were as follows: white blood cell count, 5820/μL (neutrophil 45.5%, eosinophil 4.1%, and lymphocyte 42.1%); red blood cell count, 473×104/μL; hemoglobin, 14.5 g/dL; platelet, 25.5×104/μL; sodium, 139 mmol/L; potassium, 3.9 mmol/L; chloride, 104 mmol/L. Renal and liver function were within normal range. Hormone levels were as follows: adrenocorticotropic hormone, 495.0 pg/mL; cortisol, 27.8 μg/dL; dehydroepiandrosterone sulfate, 123 μg/dL; 11-hydroxycorticosteroid, 52.0 μg/dL; estradiol, 57.9 pg/mL; progesterone, 5.04 ng/mL; luteinizing hormone, 3.35 mIU/mL; follicle stimulating hormone, 4.92 mIU/mL; free testosterone, 19.9 pg/mL; plasma renin activity, 5.7 ng/mL/hr; plasma aldosterone concentration, <4.0 pg/mL. It is noted here that she often forgot to take her medications. 

**Table 1:** Laboratory data of the subject treated with glucocorticoid and mineralocorticoid replacement therapy, compared to our hospital reference ranges. AST: aspartate aminotransferase; ALT: alanine aminotransferase; LDH: lactate dehydrogenase; ALP: alkaline phosphatase; γ-GTP: gamma-glutamyl transpeptidase; BUN: blood urea nitrogen; LDL cholesterol: low-density lipoprotein cholesterol; HDL cholesterol: high-density lipoprotein cholesterol; ACTH: adrenocorticotropic hormone; DHEA-S: dehydroepiandrosterone sulfate; LH: luteinizing hormone; FSH: follicle-stimulating hormone

Variable	Result	Reference range
Peripheral blood		
White blood cells (/μL)	5820	3300 – 8600
Neutrophil (%)	45.5↓	52.0 – 80.0
Eosinophil (%)	4.1	1.0 – 5.0
Lymphocyte (%)	42.1↑	20.0 – 40.0
Red blood cells (×10^4^/μL)	473	386 – 492
Hemoglobin (g/dL)	14.5	11.6– 14.8
Hematocrit (%)	41.3	35.1 – 44.4
Platelets (×10^4^/μL)	25.5	15.8 – 34.8
Blood biochemistry		
Total protein (g/dL)	7.1	6.6 – 8.1
Albumin (g/dL)	4.6	4.1 – 5.1
Globulin (g/dL)	2.5	2.2 – 3.4
Total bilirubin (mg/dL)	1.3	0.4 – 1.5
AST (U/L)	14	13 – 30
ALT (U/L)	12	7 – 23
LDH (U/L)	139	124 – 222
ALP (U/L)	68	38 – 113
γ-GTP (U/L)	10	9 – 32
BUN (mg/dL)	21↑	8 – 20
Creatinine (mg/dL)	1.00↑	0.46 – 0.97
Cholinesterase (U/L)	370	201 – 421
Uric acid (mg/dL)	4.6	2.6 – 5.5
Creatine kinase (U/L)	45	41 – 153
Amylase (μg/dL)	74	44 – 132
Electrolytes		
Sodium (mmol/L)	139	138 – 145
Potassium (mmol/L)	3.9	3.6 – 4.8
Chloride (mmol/L)	104	101 – 108
Diabetes and dyslipidemia markers		
Plasma glucose (mg/dL)	97	
Hemoglobin A1c (%)	5.0	4.9 – 6.0
Total cholesterol (mg/dL)	190	142 – 248
LDL cholesterol (mg/dL)	114	65 – 139
HDL cholesterol (mg/dL)	63	40 – 103
Triglyceride (mg/dL)	54	30 – 149
Endocrine marker		
ACTH (pg/mL)	495.0↑	7.2 – 63.3
Cortisol (μg/dL)	27.8↑	4.5 – 21.1
DHEA-S (μg/dL)	123	146 – 336
11-hydroxycorticosteroid (μg/dL)	52.0↑	7.0 – 23.0
LH (mIU/mL)	3.35	1.4 – 15
FSH (mIU/mL)	4.92	3 – 10
Estradiol (E2) (pg/mL)	57.9	28.8 – 525.9
Progesterone (ng/mL)	5.04↑	0.1 – 0.4
Free testosterone (pg/mL)	19.9↑	0.4 – 2.3
Plasma renin activity (ng/mL/h)	5.7↑	0.2 – 4.1
Plasma aldosterone (pg/mL)	<4.0↓	4.0 – 82.1

**Figure 1:**
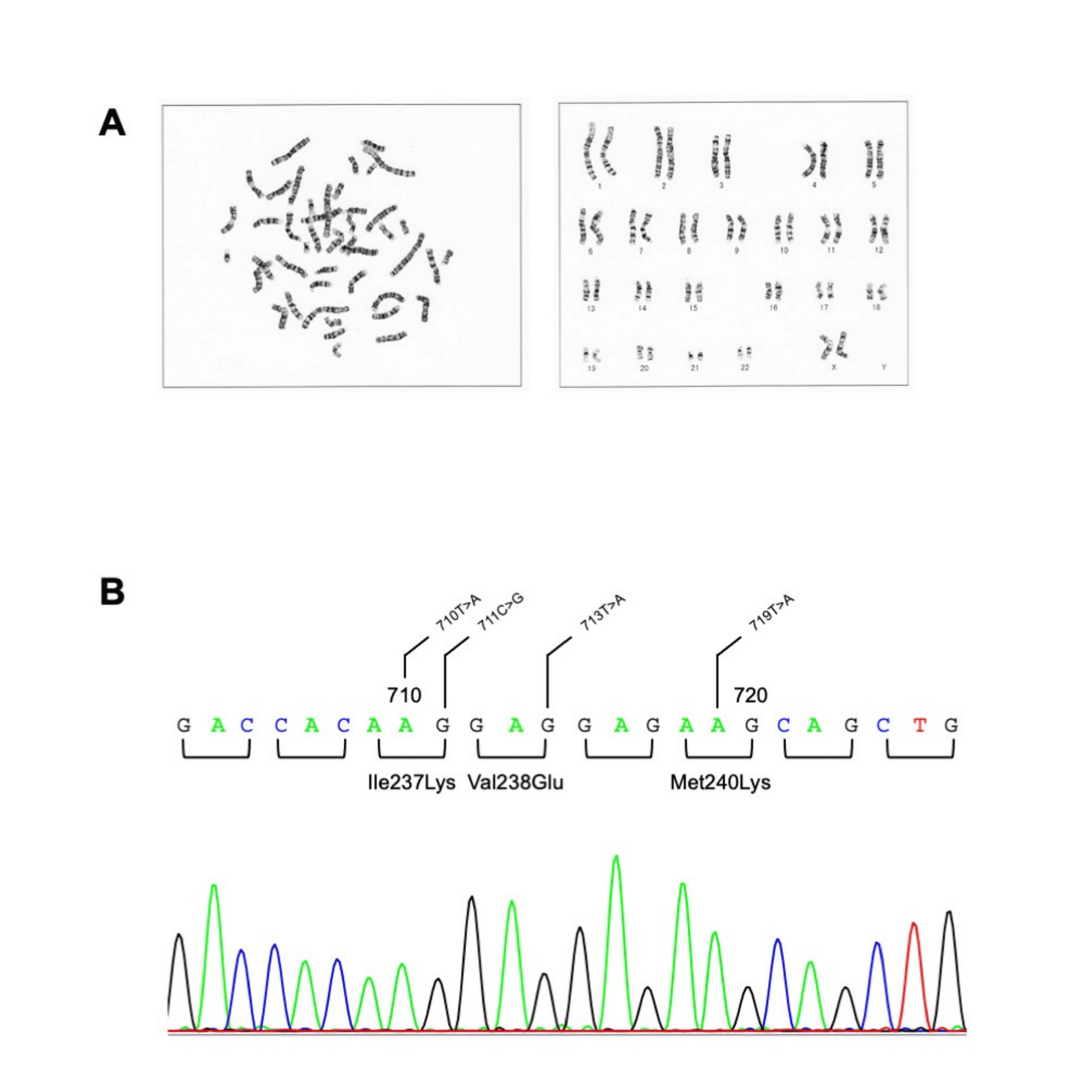
21-OHD CYP21A2 analysis A: G-band staining method, the patient's karyotype findings were human female karyotype; 46,XX; B: 21-OHD CYP21A2 analysis, a three-missense pathogenic variant was detected: p.[Ile237Lys; Val238Glu; Met240Lys]. 21-OHD: 21-hydroxylase deficiency

We encouraged her to take the medication properly and continued her treatment. She was initially thought to have an SV type of 21-OHD because of ambiguous genitalia; however, she was diagnosed with an SW type of 21-OHD and treated with hydrocortisone and fludrocortisone. Since the SW type is the most severe, but the SV type is less severe, and she had not had any genetic testing, we performed 21-OHD CYP21A2 analyzing after obtaining the approval of the individual. We examined her blood karyotype (46 XX) (Figure 1A: G-band staining method (SRL Inc., Tokyo)). A three-missense pathogenic variant (p.[Ile237Lys; Val238Glu; Met240Lys]) was detected in the CYP21A2 gene (Figure 1B: 21-OHD CYP21A2 analysis (LSI Medience Corporation, Tokyo). To the best of our knowledge, this mutation has never been registered before, but it is similar to a previously reported mutation p.[Ile237Asn; Val238Glu; Met239Lys] (NCBI Reference SNP ID: rs 786204728) [13], which was a cluster mutation found in a patient with a severe SW form of 21-OHD resulting in no detectable enzyme activity.

## Discussion

Here, we report a case of 21-OHD due to a new variant of cluster mutation of p.[Ile237Lys; Val238Glu; Met240Lys] on the CYP21A2 gene, located in the long arm of chromosome 6 in a Japanese woman. In subjects with 21-OHD, it is important to understand the correct gene mutation, because the treatment strategy depends on the degree of residual activity of 21-hydroxylase. Generally, the SW form of 21-OHD is the most severe because the hormone activity is extremely low or deficient. This patient was diagnosed with 21-OHD based on various symptoms and elevated 17α-hydroxyprogesterone levels, and we immediately started glucocorticoid and mineralocorticoid replacement treatment [14]. For this reason, she had not had any genetic tests. Despite more than 300 CYP21A2 mutations that are known to occur in 21-OHD, almost all CYP21A2 mutations appear because of gene deletions, large gene conversions, and some pseudogene-derived point mutations that ablate enzyme activity [15-17].

The close proximity between CYP21A2 and CYP21A1, which is a 98% identical pseudogene [18] (30 kilobases), appears to generate frequent mutations in CYP21A2 by two mechanisms. A mutation is an unequal crossing-over during meiosis, resulting in a complete deletion of CYP21A2, and another is a gene conversion event that results in the transfer of mutations from CYP21A1 to CYP21A2 [19]. These mutations include deletions, frameshifts, nonsense, missense, and intron splicing mutations [10]. Whereas these mutations completely stop the synthesis of a functional enzyme and are associated with SW disease, different missense mutations causing functionally important amino acid substitutions may be associated with different phenotypic forms of 21-OHD. 

A three-missense pathogenic variant in the CYP21A2 gene was previously reported as p.[Ile237Asn; Val238Glu; Met239Lys] (NCBI Reference SNP ID: rs786204728), which is the substitution of Ile to Asn at 237, Val to Glu at 238, and Met to Lys at 239 in Japanese women [13], and this mutation caused the severe SW form of 21-OHD, resulting in no detectable enzyme activity [20]. Since the three-missense pathogenic variant in this lesion caused the complete absence of the enzymatic activity, it has been well investigated. Therefore, this three-missense pathogenic variant of p.[Ile237Asn; Val238Glu; Met239Lys] is classified as “Pathogenic” on the American College of Medical Genetics and Genomics (ACMG) classification. This cluster of three-missense pathogenic variants in the G helix abolishes the enzymatic activity, possibly by interference with substrate binding [18,19]. Interestingly, in spite of such an important lesion, previously no other three-missense pathogenic variant in the CYP21A2 gene at the same lesion has been reported before, except for p.[Ile237Asn; Val238Glu; Met239Lys]. In the present case, also Japanese, we report the three-missense pathogenic variant, which differs compared to the previous report of a three-missense pathogenic variant in the CYP21A2 gene [13]. The results of our case also show and remind us once again that three-missense pathogenic variants in the CYP21A2 gene of p.[Ile237Asn or Lys; Val238Glu; Met239Lys or Met240Lys] are important lesions of the enzyme activity of 21‑hydroxylase and cause the onset of 21-OHD. Therefore, it is considered that this p.[Ile237 Lys; Val238Glu; Met240Lys] mutation is classified as pathogenic strong (ps1) on the ACMG classification [20].

There is a limitation in this case report. For the transition from childhood to adulthood in YASHCN, the symptoms and laboratory findings at the time of diagnosis are unknown. In addition, although in many hospitals and centers, genetic testing is routinely performed on patients diagnosed with 21-OHD today, it is unclear whether genetic testing was done correctly a couple of decades ago. Currently, since genetic testing is often done on a commercial basis, among physicians, there are many uncertain factors about the significance of genetic abnormalities. In this study as well, we performed the genetic analysis on a commercial basis without using the in-silico tools for variant classification. Therefore, we failed to provide the information about minimum values, indicating the damaging/pathogenic nature of variants. However, we believe that it is important for clinicians to know this subject, and we think it is important that correct genetic diagnoses are performed in young women in the fertile period, if it is not clear that correct genetic diagnoses of 21-OHD are performed.

## Conclusions

We should bear in mind the possibility that 21-OHD patients did not undergo any genetic testing because they were diagnosed with symptoms and elevated 17α-hydroxyprogesterone levels at birth. Additionally, since the genetic test may also be helpful to determine the type and severity of 21-hydroxylase deficiency, diseases such as 21-OHD may need attention and correct diagnosis with a genetic test when transitioning from childhood to adulthood in the YASHCN. Moreover, this case proves that a three-missense pathogenic variant in the CYP21A2 gene as p.[Ile237Lys;Val238Glu;Met240Lys], brings about an SW form of 21-OHD. We think that the information, including genomic, in this case report would be useful for evaluating 21-OHD in the future.
